# Acceptability and feasibility of malaria prophylaxis for forest goers: findings from a qualitative study in Cambodia

**DOI:** 10.1186/s12936-021-03983-w

**Published:** 2021-11-25

**Authors:** Monnaphat Jongdeepaisal, Mom Ean, Chhoeun Heng, Thoek Buntau, Rupam Tripura, James J. Callery, Thomas J. Peto, Franca Conradis-Jansen, Lorenz von Seidlein, Panarasri Khonputsa, Kulchada Pongsoipetch, Ung Soviet, Siv Sovannaroth, Christopher Pell, Richard J. Maude

**Affiliations:** 1 Mahidol-Oxford Tropical Medicine Research Unit, Faculty of Tropical Medicine, Mahidol University, Bangkok, Thailand; 2 Centre for Tropical Medicine and Global Health, Nuffield Department of Medicine, University of Oxford, Oxford, UK; 3 Provincial Health Department, Stung Treng, Stung Treng Cambodia; 4 National Center for Parasitology, Entomology and Malaria Control, Phnom Penh, Cambodia; 5 Amsterdam Institute for Global Health and Development (AIGHD), Amsterdam, The Netherlands; 6 Department of Global Health, Amsterdam University Medical Centers - Location Academic Medical Center, Amsterdam, The Netherlands; 7 Centre for Social Science and Global Health, University of Amsterdam, Amsterdam, The Netherlands; 8 Harvard TH Chan School of Public Health, Harvard University, Boston, USA; 9 The Open University, Milton Keynes, UK

**Keywords:** Forest goer, Malaria, Prophylaxis, Acceptability, Feasibility

## Abstract

**Background:**

In the Greater Mekong Subregion, adults are at highest risk for malaria, particularly those who visit forests. The absence of effective vector control strategies and limited periods of exposure during forest visits suggest that chemoprophylaxis could be an appropriate strategy to protect forest goers against malaria.

**Methods:**

Alongside a clinical trial of anti-malarial chemoprophylaxis in northern Cambodia, qualitative research was conducted, including in-depth interviews and observation, to explore the acceptability of malaria prophylaxis for forest goers, the implementation opportunities, and challenges of this strategy.

**Results:**

Prophylaxis with artemether–lumefantrine for forest goers was found to be acceptable under trial conditions. Three factors played a major role: the community’s awareness and perception of the effectiveness of prophylaxis, their trust in the provider, and malaria as a local health concern. The findings highlight how uptake and adherence to prophylaxis are influenced by the perceived balance between benefits and burden of anti-malarials which are modulated by the seasonality of forest visits and its influence on malaria risk.

**Conclusions:**

The implementation of anti-malarial prophylaxis needs to consider how the preventive medication can be incorporated into existing vector-control measures, malaria testing and treatment services. The next step in the roll out of anti-malarial prophylaxis for forest visitors will require support from local health workers.

**Supplementary Information:**

The online version contains supplementary material available at 10.1186/s12936-021-03983-w.

## Background

Over the past 20 years, the Greater Mekong Subregion (GMS) has recorded a substantial reduction in malaria incidence and related mortality [1]. Progress has been made through the successful implementation of various disease prevention and control measures, including increasing coverage of appropriate vector control, early case detection and access to effective anti-malarial treatments [2]. However, malaria remains a serious threat to public health, with residual transmission continuing in forested areas and along international borders [3–5], where forest goers and mobile populations are at an increased risk [6, 7]. The emergence and spread of resistance to first-line anti-malarials, including artemisinin-based combination therapy (ACT), in the GMS has revitalized elimination efforts in the region.

Forested zones are an important setting for continued malaria transmission with shade, humidity, protected mosquito breeding pools, and absence of infrastructure. The effectiveness of key vector control measures, such as insecticide-treated mosquito nets (ITNs), coils and other repellents or barriers (including long-sleeved clothes) is limited in forest settings; vectors are diurnal, bite outdoors, and workers are often engaged in hard physical labour at all hours [8–10]. Given these challenges, researchers have suggested that malaria prophylaxis for forest goers might have a potential role in prevention, control and elimination efforts [9–11].

Anti-malarials have been used prophylactically in a variety of epidemiological settings and for several at-risk groups. Intermittent preventive treatment (IPT) has been implemented and evaluated in endemic areas for pregnant women, infants and children [12–14]. Seasonal malaria chemoprevention (SMC) has been used in contexts where transmission is seasonal [15]. Mass drug administration (MDA) has a long history and recently has been evaluated in low transmission areas to accelerate elimination [16–18]. Chemoprophylaxis has been recommended for travellers and military personnel spending time in areas where transmission is likely [19, 20]. To date, malaria prophylaxis has not been widely used by adult at-risk groups living in endemic areas, such as forest goers.

The impact of anti-malarial chemoprophylaxis among forest goers on the burden of malaria is contingent on more than just its efficacy under clinical trial conditions. For all health interventions, uptake is strongly affected by their acceptability among end users. This is particularly the case for preventive interventions, such as malaria prophylaxis, for which the benefit of participation is often less immediately apparent than for treatments [21–23]. Questions also remain about how to implement prophylaxis as part of a malaria control programme. A clinical trial in northern Cambodia has recently investigated such an approach using artemether–lumefantrine (AL) [24]. The trial provided an opportunity to examine the acceptability of prophylaxis among forest goers and to investigate the feasibility of its implementation. Drawing on qualitative research methods (in-depth interviews and observation), this article explores the acceptability of malaria prophylaxis with AL for forest goers and the implementation opportunities and challenges of this strategy. The aim is to develop recommendations for future implementation as part of malaria control programmes across the GMS. To this end, forest-going practice, forest goers’ perceived malaria risk, malaria prevention and treatment-related practices (including prophylaxis), aspects of trial implementation that influence the uptake of forest-goer malaria prophylaxis, potential challenges and opportunities for integrating prophylaxis into national malaria elimination plans are described.

## Methods

### Setting

**Fig. 1:**
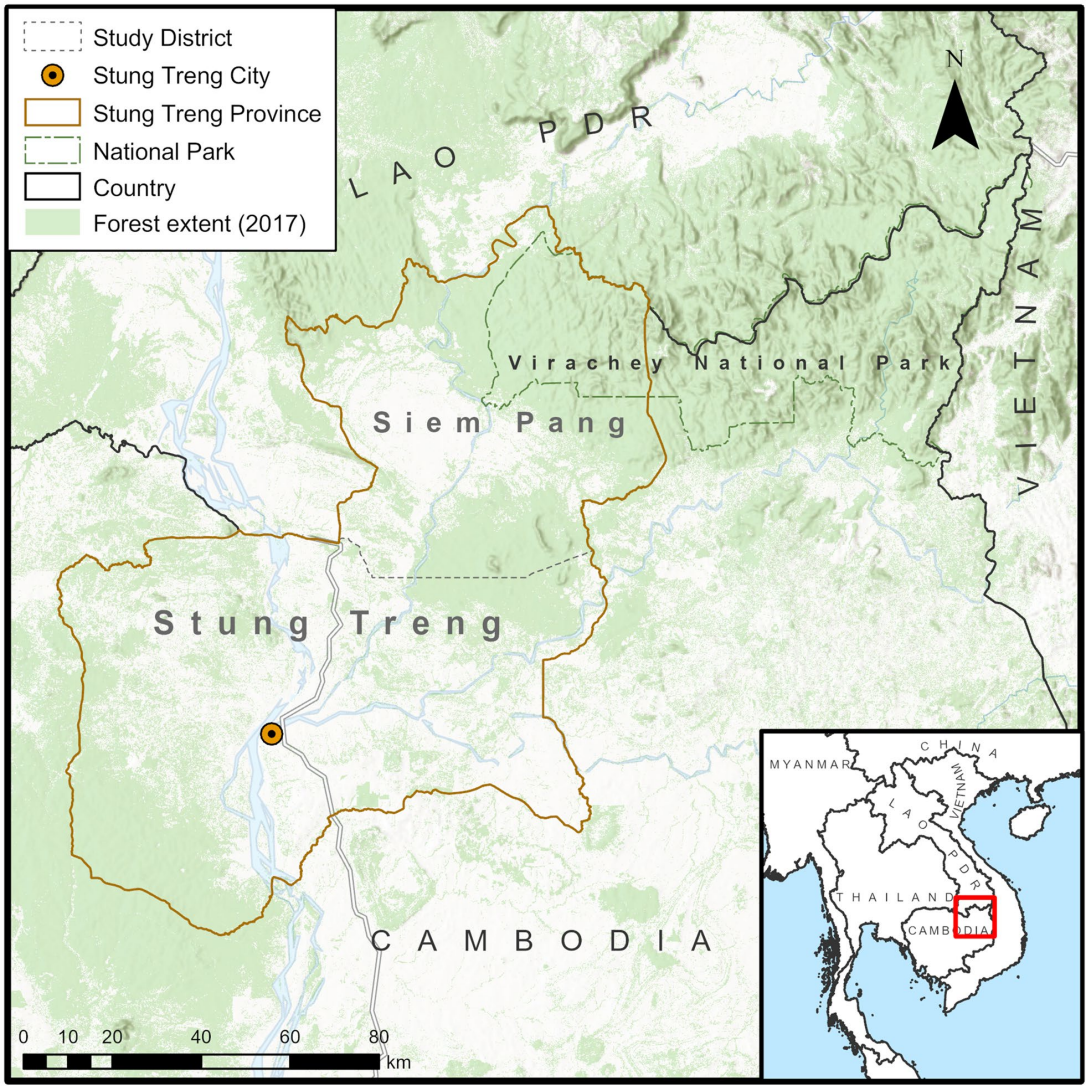
Map of study district

**Fig. 2:**
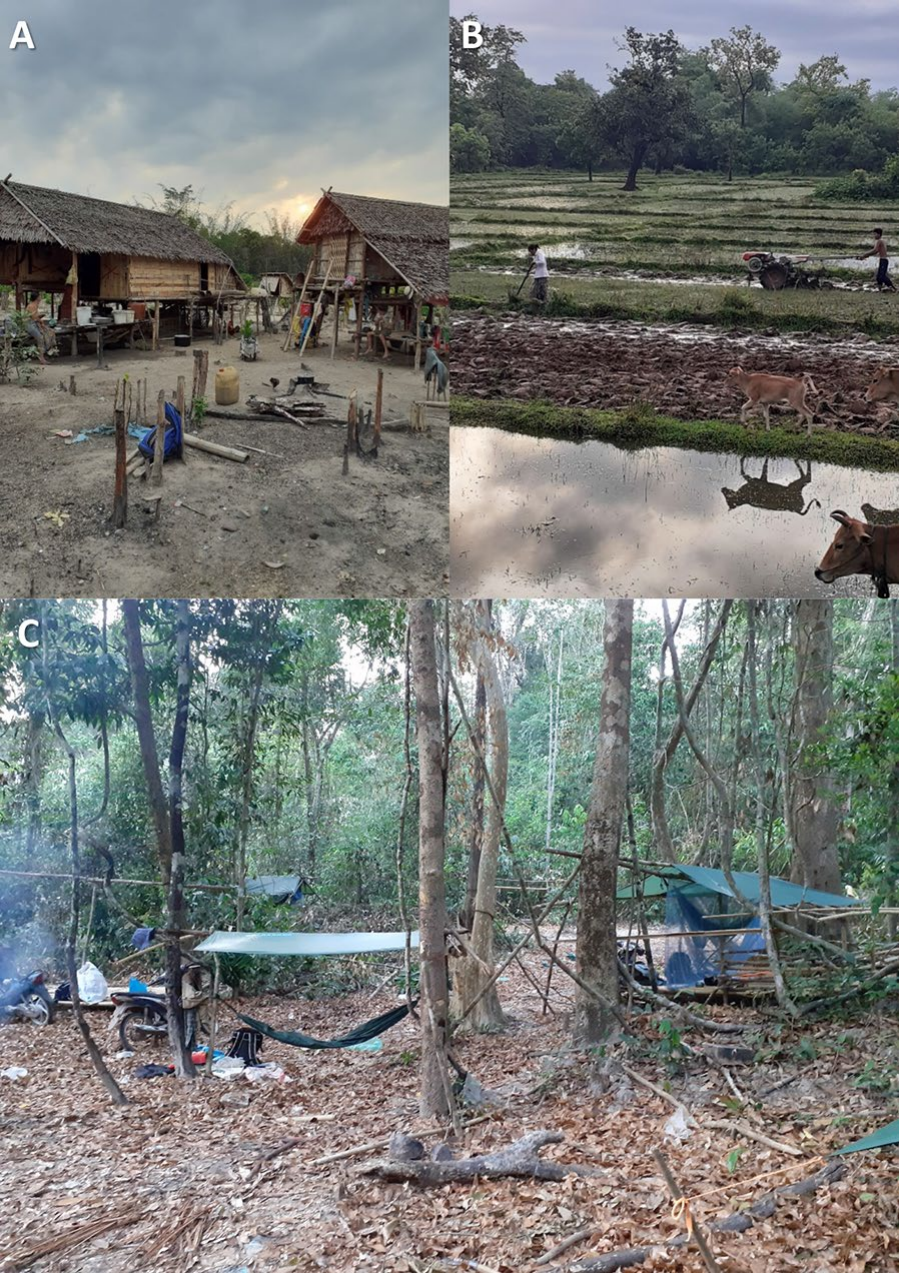
Livelihoods in Siem Pang **A** farm in the forest, **B** Rice fields, **C** logging

The study was conducted in Siem Pang District in Stung Treng Province, north-eastern Cambodia, a district which borders with Lao PDR to its north and west (Fig. 1) [25, 26]. The province is bisected by the Mekong River and is predominantly rural. The Sekong River flows through Siem Pang District; there are large forests to the north and road access to villages is restricted during the rainy season. Siem Pang has a diverse population that includes Laotian and Kavet groups. Farming is the main source of income (Fig. 2). The estimated population in Siem Pang is 24,858 with an average annual income of 800–1000 USD per person. There are two health centres in the district: Siem Pang Health Centre and Sre Sambo Health Centre. With 2151 malaria cases reported in 2019, Siem Pang district had among the highest incidence in the country (Data from Stung Treng Provincial Health Department on Malaria situation in Siem Pang, Stueng Treng, as of February 2020).

In Cambodia, malaria cases are caused by *Plasmodium falciparum*, *Plasmodium vivax*, or a mixed infection, with *P. falciparum* being predominant among confirmed malaria cases until 2011 [27]. *Plasmodium vivax* has not experienced the same decline and is now the dominant species, accounting for 85% of the malaria cases in 2019. Evidence to date also suggests that low density and asymptomatic infections are an important source of malaria transmission in the region [28]. *Anopheles minimus* and *Anopheles dirus* are important mosquito vectors of malaria in Cambodia particularly in forest and forest fringe areas in hilly or mountainous areas, cultivated forests, and rubber plantations [27] and along international borders. In 2014, 58% of the population, or approximately 8.6 million people, were estimated to live in malaria risk areas, where transmission occurs primarily in the hot and rainy season between July and November [29]. Important elimination interventions used across endemic areas include testing and treatment by village malaria workers, use of LLINs and targeting at-risk populations with 10,422 forest packs, including LLINs and LLIHNs, reported to be distributed to forest goers in 2019 [27].

The study was part of the clinical trial evaluating the efficacy of anti-malarials for prophylaxis (ClinicalTrials.gov Identifier: NCT04041973 [24]), based at the Siem Pang Health Centre. This trial randomized forest goers without clinical malaria at baseline to either artemether–lumefantrine (AL, brand name Coartem®) or a multivitamin. AL is the most widely used first-line anti-malarial treatment worldwide and is very well tolerated with an excellent toxicity profile. In Cambodia, AL has shown high efficacy [27], but it is not used routinely for for treatment. It must be taken with fat to maximise absorption of lumefantrine. The regimen was begun with a full 3-day course followed by two doses once weekly continuously for one, two or three months depending on how long they continued to return to the forest. The clinical trial took place from February 2020 to April 2021 [30]. Data collection for the qualitative study was conducted throughout the trial.

### Respondents

In-depth interviews (IDIs) were conducted with trial participants who reported visiting forested areas on more than 14 days/year. During follow-up visits for the trial, potential respondents were approached by members of a community engagement team attached to the clinical trial to enrol them in this study. Consenting participants were asked about forest-related activities and prophylaxis. Interviews were also conducted with forest goers aged 16 years or above who were not recruited in the trial due to trial ineligibility: individuals with planned pregnancy, history of allergy to anti-malarials, or cardiac conduction problems. This was done to see if their views differed from those with direct experience of prophylaxis. Recruitment took place in villages and the health centre in Siem Pang. Additional in-depth interviews were conducted with local healthcare workers, community leaders, policymakers, and the trial staff in the village, at their place of work, or via an online video/audio call (when COVID-19 control measures prevented face to face interviews).

The study was approved by the Oxford Tropical Research Ethics Committee (534-19) and Cambodian National Ethics Committee on Health Research (008). All respondents provided informed consent to participate in the study and for the interviews to be audio-recorded. Written informed consent was obtained from all study participants when they were recruited in their villages or at Siem Pang Health Centre, and verbally confirmed before the interviews were conducted several weeks later during the trial follow-up period. For other stakeholders, written informed consent was obtained before the interviews at their places of work.

Local community members and authorities were engaged from an early stage of the trial. Before beginning data collection, a large orientation meeting, smaller community meetings, and meetings with groups of forest goers were held to explain about the trial activities and their purpose. There was regular contact and communication between the study team, local health workers, as well as commune, district, and provincial level leaders.

### Data collection

Data collection tools and in-depth interview guides for each type of respondent were developed based on the initial topics drawn from a recent qualitative study on forest going and malaria-related risk in Cambodia [9]. The questions on acceptability of prophylaxis were designed based on a theoretical framework on acceptability of health interventions comprising: affective attitude, burden, ethicality, intervention coherence, opportunity costs, perceived effectiveness, and self-efficacy (Table 1) [31]. Questions on the implementation of the intervention were designed based on the Measurement Instrument for Determinants of Innovations [32] framework to capture the multi-level contexts of implementation determinants: socio-political context, health system context, health worker characteristics, and client characteristics [33].

**Table 1:** Key definitions for each component of the theoretical framework of acceptability

Affective attitude	How an individual feels about the intervention
Burden	The perceived amount of effort that is required to participate in the intervention
Ethicality	The extent to which the intervention has good fit with an individual’s value system
Intervention coherence	The extent to which an individual understands the intervention and how it works
Opportunity costs	The extent to which benefits, profits, or values must be given up to engage in the intervention
Perceived effectiveness	The extent to which the intervention is perceived as likely to achieve its purpose
Self-efficacy	An individual’s confidence that they can perform the behaviour(s) required to participate in the intervention

Adapted from Sekhon et al. [31]

IDI guides for each group of interviewees (see Additional files 1, 2, 3, 4, 5 and 6) were initially designed in English and translated by a native Khmer speaker and field researcher. The guides included key topic areas and lists of suggested questions and were designed to be used in a flexible and iterative manner: interviewers would be reactive to the responses and probe or ask follow-up questions to elicit the information on the specific topic emerging during the interviews. During development, the translations of the topic areas and suggested questions were discussed and checked with the team who were also trained on how to use the guide. The guide was then piloted with the first recruited study participants to check for any miscommunication and revised as necessary.

Interviews took place in villages, typically at participants’ homes or communal places, or at the health centre. Respondents were interviewed by one of two trained field researchers fluent in Khmer and Lao. Stakeholders were interviewed by a social scientist in English, with live translation to Khmer by one of the two field researchers.

### Observation

Observation data of the trial was recorded in an implementation diary in which the study team took field notes from observing the trial activities throughout the trial implementation. The study team had regular debrief meetings to review these notes. The field notes were imported and coded on NVivo for thematic analysis along with the interview data. The implementation diary was designed to record observation data about the implementation of the trial that may not have been captured from the interviews with trial staff and other respondents. The notes included details about trial activities. For example, questions that were raised by community members during community meetings, and challenges that occurred during recruitment or follow-up activities in the villages.

### Data processing and analysis

After respondents gave their consent, interviews were audio-recorded and subsequently transcribed and translated to English by field researchers. The translated transcripts and field notes were imported into NVivo version 12 (QSR International Australia) for qualitative thematic analysis. All transcripts were read several times and coded line-by-line using inductive and deductive approaches: the codebook used was initially based on the main research topics. Subsequently, during the process of coding, themes that emerged from the data were incorporated into the codebook.

## Results

### Demographic characteristics of respondents

The findings presented are based on individual in-depth interviews with 27 forest goers including 23 trial participants and 4 non-trial participants. A further 19 interviews were conducted with healthcare workers, community leaders, policymakers, and trial staff. Characteristics of the respondents are summarized in Table 2. The findings were also informed by analysis of the observations during implementation of the trial carried out at the site and recorded as field notes by members of the trial team. It was found that activities and malaria-related experiences of forest goers were similar irrespective of whether they participated in the trial; while male respondents reported being more mobile and engaged in multiple forest activities than their female counterparts.

**Table 2:** Demographic characteristics of respondents

*Forest goers (Trial participant and non-trial participant)*	
Sex	
Female	5
Male	22
Age (years)	
16–20	3
21–30	9
31–40	6
41–50	6
51–60	3
Number of household members	
1–4	4
5–8	17
9–13	6
Years of education	
0	6
1–6	15
7–12	6
First language	
Khmer	12
Lao	7
Kavet	8
*Stakeholders and trial staff*	
Sex	
Female	4
Male	15
Age (years)	
16–20	0
21–30	4
31–40	5
41–50	6
51–60	4
Type of respondents	
Healthcare workers	7
Community leaders	5
Policymakers	3
Trial staff	4

### Forest goers and forest visits

#### Environment

In Siem Pang, forest-going and farming were described as important livelihood activities (Fig. 2) and the main source of income. Farms are usually located at the forest fringes on the edge of the forest or close to the villages. Rice, cassava, and cashew nuts were the main agricultural crops. Forest goers reported engaging in various forest activities including hunting, logging, fishing, and collecting forest products, such as mushrooms, honey and bee’s nests, wild orchids, resin, and particularly malva nuts (Samrong). Respondents described travelling to the forest by foot, boat, motorcycle, or tractor (“machine cow”); the latter was described as crucial to transport their supplies to the forest and for bringing out forest/farming products on return. Forest goers are predominantly male and engaged in labour-intensive work in the forest. Respondents described visiting the forest usually with their male family members or friends, except when collecting Samrong for which a whole family with females and children would often join. Apart from local residents who regularly visit the forest, forest rangers and military officials occasionally patrolled in groups with permanent campsites in the forested areas.

#### Seasonality

Collecting forest products was described as a year-round activity, with forest goers collecting different products in different seasons. Respondents mentioned visiting the forest on a regular basis when they have time off from their farm work, and especially when they combined forest visits with finding food in the forest: “tov rok luy, tov rok sad, tov rok banle” (“make money, find animal and vegetable”) was a common response when forest goers explained why they went to the forest. Respondents also described the harvest season as the busiest time in their farm during which some made fewer trips to the forest and usually spent nights at small farmhouses in rice fields or farms with their family members. Farmhouses were often described as a hut; some with a roof and walls made of tree branches and leaves and some without walls (Fig. 2a). After the harvest season, most respondents would stay in their home village, whiles some male family members described going back to the forest or looking for a temporary job, such as working in plantations, in Stung Treng or in nearby provinces.

#### Forest visits

Respondents described their resting places as make-shift camps, the details of which depended on the size of the group. Forest goers commonly slept in hammocks under trees or under a tent built using a rubber cloth or tarpaulin. The locations for setting up a sleeping place were often in a clear, flat area and/or by a stream or water source in the forest so they could use the water for cooking or washing. The length of forest visits usually depended on the nature of each activity in the forest, ranging from a few days to weeks, for example, hunting and tracking animals, or finding and collecting enough Samrong for sale. They also described other factors such as running out of food or getting sick in the forest. One respondent mentioned travelling back and forth to the forest to get more food from the village for the group members during their forest visits. Nine respondents mentioned logging and collecting wood to build houses in the village or to sell, however participants did not discuss the activity and its whereabouts in detail during interviews. During follow-up visits, staff observed that villagers discreetly engaged in logging activities and avoided disclosing the locations in the forest for fear of being punished by the authorities.

Respondents recognized the risk of malaria from their forest visits: some described that the risk was anywhere in the forest, others specified names and descriptions of particular hotspots, such as mountains, streams, or areas with certain kinds of trees such as bamboo in the forest. Some respondents also associated those places with the presence of mosquitoes. One described past malaria infections as unavoidable with the limited protection available, for example when they were urinating in the forest, mosquitoe bites were unavoidable. A few respondents also felt that they were at risk at their farms describing the environment as having a lot of mosquitoes, trees, and rubbish.

*Interviewer (I): Do you think anything you do puts you at risk of getting malaria?*

*Respondent (R): Yes, at risk because we cannot prevent 100%. Mosquitoes bite when we go to urinate in the forest. There are a lot of mosquitoes in the forest…Ta Ngoy mountain…if I go to this place I get malaria*.

IDI: Male, 39 years old, trial participant from Samor Khnong village

### Experience of malaria infection, prevention and treatment

#### Experience of multiple bouts of malaria

All respondents described having had malaria in the past, with some having had multiple bouts in a year. Most respondents described mosquito bites as the main cause of malaria, whereas some mentioned poor hygiene or hot weather as additional causes. Respondents described malaria symptoms as headache, chills, cold, sweats, fatigue, feeling hot in the chest, and muscle pain. The cycling of fever was used to distinguish malaria from other febrile illnesses. Most respondents mentioned that they had tested positive and been provided with an antimalarial by a village malaria worker (VMW) in the past. Although asymptomatic malaria was generally not recognized among forest goers, most health care workers and half of trial participants said it is possible to have malaria without symptoms from their past experience. Possible infection after mosquito bites but prior to symptoms was also recognized.

*“It’s possible because after getting mosquito bites, patient don’t have symptoms yet, so after people come back from the forest they need to do RDT.”*

IDI: Male, VMW from O’Chay village

*I: Do you think you might have malaria but do not have symptoms?*

*R: Yes, because I don’t know I have malaria in my body or not. If have mosquito bite, I can get malaria.*

IDI: Male, 39 years old, trial participant from Samor Khnong village

#### Use of multiple prevention measures

Forest goers reported using a combination of measures including long sleeved clothes, sometimes with balaclava or gloves, making fire, and sleeping under mosquito nets to prevent mosquito bites in the forest. Most respondents mentioned receiving an ITN from VMWs and/or a health centre, whereas some purchased the nets from a local market. A hammock and blanket were mentioned as an alternative for some when they did not bring the net to the forest. A few respondents mentioned using mosquito coils and fewer reported using mosquito repellents because of their higher price and availability in Siem Pang. Although no respondents described taking medicine to prevent malaria prior to the study, one respondent mentioned taking paracetamol that he bought from a local market to the forest in case he got sick. One respondent reported that in the past villagers brought antimalarial medicines for self-treatment in the forest when there were signs of malaria without having a malaria test.

#### Experience of malaria testing and treatment

Respondents reported being familiar with the VMWs and/or malaria mobile workers (MMWs) in their village as the primary point of malaria testing and treatment and whose service also included referring patients to the health centre (HC) in Siem Pang. A few forest goers reported visiting the health centre for a malaria test because of additional benefits e.g. check-ups for other illnesses, trust in the HC nurses, proximity of their house to the health facility, or unavailability of VMWs or MMWs in the village. Some described a preference to visit private clinics or pharmacies because they provided a faster service than the HC. One respondent described buying anti-malarials at a clinic during the weekend for self-treatment because he needed to return to work in the forest quickly.

Side effects of past malaria treatments were frequently mentioned by forest goers. These side effects (e.g. fatigue, dizziness, vomiting, nausea) caused some to visit private clinics (for intravenous fluids) or a traditional healer. Although most did not recall or know the name of the antimalarial they had taken (in Siem Pang, antimalarial or malaria medicine is frequently referred to as “Thnam Krun Chanh” in Khmer or “Yaa Krun Chanh” in Lao), malaria workers and trial staff described that quinine, mefloquine, or artesunate-mefloquine had been provided for treatment by the VMWs and health centre staff, or private clinics and pharmacies in the past.

Policymakers and malaria workers described how VMWs and MMWs are tasked with testing up to 50 villagers per month to detect symptomatic and asymptomatic cases in their village as an active case detection strategy to eliminate *P. falciparum*. VMWs reported difficulties persuading asymptomatic individuals to be tested. Even though testing services were easily available (VMW services are based at homes in the villages, and MMWs are stationed at a health post on the roadside, at an entry way into or out of the village, or they visit the forest or farms to do active case finding), the workers described having to go and visit villagers themselves when they came back from the forest to convince them to take the test.

### Acceptability of prophylaxis

The results presented here are based on interviews with trial participants during follow-up visits after one or two months of trial drug administration, non-trial participants at baseline, as well as with stakeholders at various time points during the trial, and observations made throughout its implementation. Findings are reported based on the framework on acceptability of health interventions. It was found that awareness of the clinical trial, worries about malaria infection and its (economic) implications, and perceived effectiveness of prophylaxis contributed to the acceptability of prophylaxis.

#### Intervention coherence (awareness and understanding)

Trial participants appeared to be well aware of the purpose of the trial, the drugs, and the activities. The participants were informed about malaria risks and how to protect themselves, particularly how a person can get infected from mosquito bites, how the prophylaxis may prevent the infection, and why they should continue to protect themselves by using mosquito nets. Trial staff viewed community meetings and forest goer meetings prior to the recruitment as conducive to this awareness and understanding of malaria (i.e. cause of infection, mosquito bites) and the aim of the trial (i.e. how prophylaxis can prevent or reduce the chances of getting sick). Respondents discussed how some villagers were not informed about the trial activities in the village, mainly because they were working in their farm away from the village when the meetings took place and thus did not participate. Four non-trial participants who were ineligible to join the trial because of their health reported that they were informed about the trial by VMWs or MMWs in their village, and were interested to take anti-malarials to protect themselves from malaria if their health conditions were better.

#### Ethicality (fit with values and preferences)

Trial participants joined the trial because they were concerned about having malaria in the forest. Of all the diseases, malaria worried them the most: it was seen as a dangerous disease; potentially deadly if left untreated. Some respondents described their concerns of the infection resulted in loss of time from work, particularly those who lived in remote villages. Availability of regular health check-ups and compensation for travel cost each time participants came to a follow-up visit were part of their motivation to join the trial. Other perceived advantages from trial participation included the possibility of better care for another illness or accident (although this was not explicitly included in the trial design). For example, during enrolment, medications for some common health problems e.g. muscle pain, gastritis, or vitamin supplements, were offered to the participants who were ineligible for the trial. Blood pressure was checked for participants in high-risk groups for hypertension and those found to be hypertensive were advised to go to the health centre for medical advice. The staff observed that the programme’s provision of care was positively received by the participants, describing that they felt happy that someone was taking care of their health and well-being. Village chiefs and VMWs described being concerned about whether malaria cases might increase after the trial had finished.

*“If I take the medicine and still feel ok, then I would continue to take it. If not, then I would not take it because it would make me feel worse.” *

IDI: Male, 28 years old, trial participants from Lakay village.

#### Affective attitude

Forest goers were positive about the trial, describing trust in staff and the good reputation of the research centre. Participants often referred to trial staff as “lok krou” or “neak krou”, and “lok krou pet” or “neak krou pet” (referring to male/female teacher and doctor in Khmer; VMWs/MMWs are often referred to as “pet phum” or village doctor). Interviews with the trial staff highlighted trust and clear communication as important factors in recruiting participants. This was also crucial for explaining the medicine’s preventive value, how to take the tablets and why it is important to take them according to the instructions. Interviews with, and observations by, the staff highlighted potential participants’ questions about the drug’s efficacy during the recruitment activities. Some respondents asked during interviews whether the drug can really protect and if so for how long. Interviews with the trial staff found that the participants would like to know which trial drugs (i.e. AL or multivitamins), if any, are effective at preventing malaria.

*“The drugs are good, easy to take, no side effects ... I like it because they can protect me from disease. Someone told me that s/he stopped taking them because s/he had side effects, like feeling dizziness, feeling as if they would fall down when walking, or having blood in the stool.”*

IDI: Male, 32 years old, trial participant from Kirvongsa Krom village

#### Burden and opportunity costs

Side effects of the antimalarial were described as the main determinant for respondents to make a decision to join the trial or continue to take prophylaxis. Most respondents reported experiencing minimal side effects from the preventive medicine and continuing to work and visit the forest as usual. Some reported staying at home and working less during the first few days after taking the trial drugs due to side effects, such as dizziness, fatigue, blurred vision, nausea, and/or vomiting. The trial was open label so participants would have been able to identify which medication they were taking. Policymakers and staff were also familiar with the complaints about side effects associated with anti-malarial treatment. One non-trial participant discussed how he was willing to take the preventive medicine despite having had severe side effects from anti-malarials.

Trial staff observed that the trial participants linked the severity of side effects with the number of tablets per dose (per day), and that those side effects were reported mostly during the first and second days of the AL course. Staff also observed more complaints about side effects when the participants received “the yellow one” (i.e. AL). Some participants preferred taking “the black one” (i.e. multivitamins) because there were fewer tablets per intake/dose. Some respondents reported that they prefer to take the prophylactic once a week (taken in week 2 onwards) rather than three days consecutively (taken in week 1) as they felt less comfortable taking many pills within a short period of time. They described their concern about side effects relating to whether it would affect their work in the forest, farm, or around the house. Staff also reported that older female participants and younger participants complained more about the side effects than other groups of participants. They were reassured and (again) advised to take the medicine after a meal.

*“Some people complained, especially females were scared of side effects, didn’t want to take the drug … a few people felt dizziness after taking the drugs … especially young boys they felt uncomfortable after taking the drugs. Villagers feared malaria medication may cause side effects such as dizziness, nausea and vomiting because most of them had malaria before and had taken drugs for malaria.”*

IDI: Male, 23 years old, trial participant from Kiribas Leu village

In terms of the trial procedures, participants disliked having blood samples taken, which was viewed as harmful for their bodies or general health (“losing blood”) and that it may cause fatigue and dizziness. Trial staff described participants’ concern about the amount of blood taken for pharmacokinetic analysis (1 ml for each participant) which is more than the usual dried blood spot for a malaria test that most villagers are familiar with.

#### Perceived effectiveness

Respondents described the effectiveness of prophylaxis in terms of fewer malaria cases and better health. They observed that they were not infected with malaria during the trial. One VMW reported that participants came to them to have a malaria test because they wanted to know whether the medicine can protect them from malaria. Male and female respondents did not perceive effectiveness or acceptability of prophylaxis any differently. Nevertheless, male community members were more mobile than their female counterparts and their mobility affected their uptake of prophylaxis (taking the tablets and/or joining the trial) and attending a follow-up visit. Females’ motivation to take prophylaxis may be influenced by their pregnancy and/or contraceptive use: one female non-trial participant described that the medicine would be beneficial but did not join the trial due to her future pregnancy plan.

Trial participants whose use of other malaria prevention measures in the forest was limited were keen to continue taking prophylaxis after the trial. For example, they could usually only use bed or hammock nets, wear long-sleeved clothes, and/or make fires in the forest, whereas mosquito coils or repellents were too costly or unavailable in the village. One forest goer felt that the medicine can protect him from malaria and continued to use a mosquito net because the protection is not “100%”. This was also highlighted during interviews with healthcare providers. They described the benefits of the medicine in preventing malaria at times when villagers are engaged in forest activities.

*“In the explanation you have to mention concerns … what are the benefits for them in the future. One example is that some people cannot use the nets 100%. Sometimes when the man is drunk, they may sleep outside of the net. If we can provide the drug to them, it’s also a kind of prevention … For the forest goers, at night time, if they are going to pass stool in the jungle … or when they travel and carry the nets on their back, during when they are not protected and the mosquito can bite them.”*

IDI: Male, 50 years-old, healthcare worker in Siem Pang

Some respondents’ motivation to participate during the subsequent recruitment in later villages was affected by the perceived effectiveness of prophylaxis and decreased number of cases from the initial villages. Trial participants were also highly motivated to join the trial during the Samrong season (March–April) and rainy season (July–October) during which they travelled regularly to the forest or forest farm. Observations in the villages suggested that they were less motivated when the forest visits become less frequent during the dry season or after the harvest (December-February). The follow-up interviews with VMWs and community meetings in the village also suggested greater trial attrition and lower adherence to the trial drug at this time.

#### Self-efficacy

Trial participants reported good adherence to the trial drugs, although some mentioned missing the occasional dose because they forgot or were too busy with work. No-one reported discarding or sharing doses. Trial staff described that most participants were able to take the drugs as indicated and that the provided drug calendar was useful to remind participants to take the prophylaxis on time. However, participants needed clear explanations, particularly in their own language/dialect in some villages, to ensure that they were able to understand the written schedule. VMWs and village chiefs described the provision of prophylaxis in groups—rather than individually—as a more effective approach because group members were able to remind each other to take the drugs as instructed and to take the drugs with them on visits to the forest, which reduced the chances of missing doses. Trial staff also described the advantage of selecting a representative or a group leader of forest goers to monitor his or her group member’s drug intake.

*I: How should we explain to villagers why they should take medication when they’re not sick?*

*R: I think it will take time like I said. We also need to be patient and gentle when explaining to them … if you get sick it can get worse … working with the people in this district, sometimes they cannot read a word. What we have in our stamps [referring to the drug calendar], the symbols like for day and nighttime, what it means, they don’t even know what those mean. We need to find someone who is close to them to remind them all the time … if they do not take the medicine as prescribed it will not work, right? Because the medicines the doctor gives them, they need to take as they were told*.

IDI: Female, 39 years old, community leader in Siem Pang.

### Implementation opportunities and challenges

Interviews with trial participants and staff suggested that the community and participants were satisfied with the trial and the care they received. Respondents described the research team, VMWs, and health centre as preferred providers of prophylaxis in the future. When probed about this, respondents described clear explanation about prophylaxis and provision of (ancillary) care. Familiarity with, and proximity to, VMWs were reasons for preference for VMWs. Trial staff reported that, with additional training, VMWs would be suitable providers because they are local residents who are aware of their neighbours’ forest visit schedule and able to communicate in local/ethnic languages. A few respondents described the health centre to be a good option as a trusted provider in general. For other households, however, a private clinic was the preferred health facility because of the faster provision of services and treatment of other diseases.

Trial staff described how community meetings and stakeholder engagement contributed to cooperation of participants, particularly in the pre-recruitment period, among community members who could spread the word to forest goers who may be working away from the village in a farm or the forest. Engaging trusted members of the community, such as VWMs and/or MMWs, village chiefs, and sometimes the elderly was described as contributing to a wider provision of information in the villages. Observations by trial staff also found that conducting recruitment and follow-up visits in the village, rather than at the health centre, is a better approach to minimize missing potential participants and losing them to follow-up. Female staff reported paying special attention when recruiting female participants who would have to consult with their partners on their use of contraceptive pills and condoms, which were required in the trial protocol for females of reproductive age.

Trial staff viewed support from VMWs/MMWs in the village as crucial for communicating important messages in the local language and supporting the communication between the villagers and the trial team. In some villages where Lao is more commonly spoken, staff emphasized the importance of communicating with participants in their language, especially when explaining drug administration. In some study villages where languages such as Kavet were widely spoken and study staff were not sufficiently fluent, VMWs or MMWs who are local residents and trusted village members were asked to help. However, communication with the VMWs was sometimes challenging: using mobile phones was not easy due to poor mobile connectivity, especially in remote villages, from which the staff were informed about recent malaria cases in one village only from the VMW monthly meeting at the health centre.

Interviews with policymakers highlighted concerns about the potential effect of prophylaxis on ITN use. They raised the question whether participants might consider the protection offered by prophylaxis as sufficiently high that participants would no longer use other prevention measures, particularly ITNs. Policymakers emphasized the need for a clear and thorough explanation about this approach and how prophylaxis was only one of several ways to protect against malaria and not a replacement for nets or other vector control measures. Trial participants reported that they still used mosquito nets and described that the medicine may not entirely protect them from malaria, or that without the net they could not sleep in the forest because mosquitoes still bite and make a noise near their ears.

## Discussion

Drawing on in-depth interviews and observations, the findings of this study indicate that prophylaxis with AL for forest-goers in northern Cambodia is acceptable under trial conditions. Several findings support this conclusion: the community’s awareness and perceived effectiveness of prophylaxis, their trust in the provider, and malaria as a local health concern. Effective implementation of prophylaxis depends on addressing critical concerns by forest goers and stakeholders related to the side effects of anti-malarials, the influence of prophylaxis on other prevention practices, dealing with complex forest-going activities and access to and quality of malaria-related care.

Concerns about potential side effects and their economic implications discouraged uptake in this and previous studies. This is unsurprising given past experiences of anti-malarials (e.g. mefloquine, artesunate-mefloquine (ASMQ) [9] and has been described in regard to the administration of (preventive) anti-malarials in many contexts. For example, the perceived side effects of MDA in Battambang (Cambodia) led to lost productivity, particularly during the farming season, and additional healthcare payments (for intravenous fluids) [11, 22, 34], which discouraged participation. Pregnant women who received preventive anti-malarials (as part of IPTp) have also raised concerns about the unwanted effects of anti-malarials, such as vomiting, worries that the drugs may affect their pregnancy or children [35, 36] and the extra spending on food after taking the medicine [36]. Travellers’ anti-malarial choices were also influence by pill burden [37, 38], cost [39, 40], perceived risk, travel characteristics [38, 41, 42] and scepticism about effectiveness [42]. A recent systemic review highlighted how potential side effects influence adherence to chemoprophylaxis among travellers [43]. This highlights the importance of selecting a well-tolerated regimen for any preventive medication including anti-malarial chemoprophylaxis.

The trial team, comprised of medical doctors, nurses and health centre staff, assisted by VMWs and MMWs as the service providers, was well received by participants. The team’s track record of treating malaria patients and providing clear information about the trial strengthened the acceptability of prophylaxis [44, 45]. The findings indicate that trust in the trial team was sufficient reason to participate even if forest goers did not have a complete understanding of the prophylaxis. The roles of inter-personal and institutional trust prompting uptake of preventive anti-malarials have been highlighted elsewhere—both in clinical evaluations of interventions and as part of real-life implementation [46–50].

VMWs and MMWs were often regarded as a senior, educated, and trusted member of the villages, even though most of them had had no higher education. As described elsewhere, they were recognized as the main point of care for malaria testing and treatment in the study communities [9, 35, 51–53]. Trial staff were confident that, if provided with the necessary training, VMWs could provide prophylaxis to forest goers. With a system of monthly meetings already in place, the district health centre could also provide the necessary training and support to VMWs who are well-placed to communicate the potential side effects that participants (may) experience.

Participants were motivated to participate by concerns about malaria and the increased risk of malaria associated with forest visits. In some cases, the enthusiasm to participate was influenced by the frequency of forest visits, which varied over the course of a year due to the nature of forest activities. Some forest goers, especially men, were highly mobile and their time away from the village affected the uptake of prophylaxis (or attendance at follow-up visits with trial staff). Some looked for temporary employment in other districts or provinces, particularly after harvest or in the dry season when forest visits yield fewer wild products. Forest goers may also engage in logging activity whose illegal nature might influence their decision to participate; they may not wish to disclose the information of their forest visits, such as locations or length of stay, to unfamiliar individuals or authorities. Forest goers engaged in a variety of different activities; knowing the travel patterns of forest goers and the local community’s calendar for visiting the forest are important pieces of information to predict the likely uptake of, and adherence to, prophylaxis.

The seasonal timing of preventive therapy has been discussed with regard to the implementation of MDA in Cambodia. Mild illnesses were linked to administering anti-malarials in the rainy season [31, 54], when uptake was affected by farming obligations [55], and work-related absences from villages [38, 56, 57]. Travel has also led to missed doses of IPTc, while distance and difficulty travelling in the rainy season created barriers for staff to distribute IPTc [58]. For IPTc, respondents seemed positive about home-based delivery [54, 59], whereas in some cases respondents expressed concerns about caregivers’ ability to administer drugs at home [58]. Given their residence in, and proximity to, the community, VMWs are aware of the timing of forest-going and hence are well placed to be the provider of prophylaxis to forest goers.

These findings indicate that the respondents continued to use malaria prevention measures they had been using before they joined the trial. This may allay some of the concerns policy makers expressed about prophylaxis competing with ITN use. This may have resulted from the emphasis that trial staff placed on continued ITN/hammock net use during the engagement activities (e.g. community meetings) and enrolment. Furthermore, even when taking prophylaxis, bites from the vectors often remained a nuisance. The prevention provided by anti-malarials, has often been viewed as incomplete: the parents of infants who received IPTi did not view this (even vaccinations) as full protection; similarly, caregivers of children who received IPTc, did not perceive the medication as a substitute for bed net usage [58].

The findings from this study can be summarized as four main implications for the intervention (see Table 3). Ideally any future implementation of prophylaxis for forest goers in real-life circumstances should be accompanied by simultaneous evaluation of its impact on other prevention practices. Communication strategies should continue to frame preventive anti-malarials as part of a package of interventions, emphasizing the need for each intervention. Combining the use of anti-malarials and ITNs was suggested to optimize elimination strategies in the context of multi-drug antimalarial resistance [60]. A recent study also suggested that IPT for forest goers should be considered as a strategy given the ongoing low-density transmission among this group [61]. However, this would generally involve less frequent dosing than prophylaxis with consequent gaps in protection. To strengthen the use of prevention measures, prophylaxis could be provided as part of a set of interventions tailored for needs of forest goers, adding to existing local malaria services such as intermittent screening, distribution of forest packages, early diagnosis and effective treatment.

**Table 3:** Main implications for implementation of malaria prophylaxis among forest goers

Main policy implications	
Prophylaxis as a part of malaria intervention	Prophylaxis should be implemented in combination with existing malaria services including use of ITNs, distribution of forest packages, and prompt testing and treatment Targeting forest goers should consider their travel patterns and seasonality to ensure coverage of and access to the intervention, particularly among mobile population groups
Choice of regimen	Choice of regimen needs to consider frequency, dosing, and especially potential side effects of the drug to encourage uptake and minimize non-adherence
Delivery of prophylaxis and provider	Delivery of prophylaxis should be from a local, trusted, and trained provider with support from an equipped healthcare facility Prophylaxis should be prescribed with a package of high-fat food (a pack of biscuits was used in this trial) to maximize lumefantrine absorption; the package should be easily portable and convenient for travelling and consumption in forest settings where meal preparation may be difficult
Messages about prophylaxis	Messages about prophylaxis should be clear and comprehensible (verbally and/or visually) in local language(s) with considerations for illiterate individuals and ethnic groups Information about the cause of malaria infection, how prophylaxis works as a prevention, and why some malaria patients are asymptomatic can create a better understanding of prophylaxis and encourage its uptake Emphasize the importance of continuing other modes of prevention to protect from mosquito bites (i.e. use of other measures) together with prophylaxis (i.e. preventive medicine) Convey that side effects are rare and mild, and short-lived for those who may experience them

## Strengths and limitations

This is the first study that has used qualitative research methods to specifically address acceptability and feasibility of malaria prophylaxis among forest goers in the GMS. Using a team of three trained researchers to collect data guarded against the undue influence of a single data collector on the findings. The findings are mainly drawn from reported data and might be subject to desirability bias, however, observations and informal conversations provided additional information on the context of trial implementation and on sensitive topics, such as logging. The interviews were conducted in Khmer and Lao which are the spoken languages in the participants’ daily lives. Interviews with stakeholders were conducted in English with translation by a local team member when necessary; some were conducted via an online call due to travel restrictions during the COVID-19 pandemic. Most respondents were male, which reflects the population of forest goers and those at highest risk for malaria, and were ethnically diverse, drawn from a range of villages. The findings from interviews with trial participants which may have been from a biased group of forest goers who were willing to participate in a clinical trial and received intensive education and support introducing potential lack of external validity. This made it difficult to separate out views specifically about prophylaxis from views about participating in the clinical trial. The participants were therefore supplemented by interviews with those ineligible to join the trial, the number of which was very low. The framework developed by Sekhon and colleagues [31] was useful in exploring the multi-faceted nature of acceptability, it was however difficult to distinguish opportunity costs from burden of the intervention and they were hence merged in the findings.

## Conclusion

The acceptability of anti-malarial chemoprophylaxis among forest goers in northern Cambodia is contingent on awareness of and concern about malaria, trust in the provider, and how prophylaxis is explained and delivered. The findings highlight how uptake and adherence to prophylaxis are influenced by the balance between perceived benefits and burden of anti-malarials, particularly their effectiveness and side effects, and the seasonality of forest visits and its influence on malaria risk. Implementing prophylaxis needs to consider how the preventive medication could be incorporated into an existing package of interventions of vector-control measures and malaria testing and treatment services; engaging multi-level stakeholders; and strengthening VMW’s capacity. The next step in the roll-out of forest goer malaria prophylaxis should be to explore how local health workers perform in managing prophylaxis outside of a clinical trial, and work out how best to target and scale up prophylaxis in the region.

## Supplementary Information

**Additional file 1.** Trial participant IDI guide.

**Additional file 2.** Non-trial participant IDI guide.

**Additional file 3.** Trial staff IDI guide.

**Additional file 4.** Healthcare worker IDI guide.

**Additional file 5.** Community leader IDI guide.

**Additional file 6.** Policymaker IDI guide.

## Data Availability

The data on which this article is based cannot be shared publicly due to confidentiality of the individuals who participated in the study. The data are available upon reasonable request to the Mahidol Oxford Tropical Medicine Research Unit Data Access Committee (datasharing@tropmedres.ac) complying with the data access policy (https://www.tropmedres.ac/units/moru-bangkok/bioethics-engagement/data-sharing/moru-tropical-network-policy-on-sharing-data-and-other-outputs) for researchers who meet the criteria for access to confidential data.

## Abbreviations

ACT: Artemisinin-based combination therapy
AL: Artemether lumefantrine
ASMQ: Artesunate mefloquine
GMS: Greater Mekong Subregion
IDI: Individual in-depth interview
IPT: Intermittent preventive treatment
IRS: Indoor residual spraying
ITN: Insecticide-treated mosquito nets
MDA: Mass drug administration
MIDI: Measurement instrument for determinants of innovations
MMW: Mobile malaria worker
RDT: Rapid diagnostic test
SMC: Seasonal malaria chemoprevention
VMW: Village malaria worker
