# Supplementary material for: Acceptability and feasibility of malaria prophylaxis for forest goers: findings from a qualitative study in Cambodia
Source: Malar J. 2021 Nov 25;20:446. doi: 10.1186/s12936-021-03983-w (PMC8613728; doi:10.1186/s12936-021-03983-w)
Supplement: Supplementary file 1 — Additional file 1. Trial participant IDI guide. [file 12936_2021_3983_MOESM1_ESM.docx]

**Interview guide for PAL trial participants**

| **Instructions:**   - Follow the informed consent procedures - If consent is given, audio record the interview - This interview guide is to be used in a flexible manner. - The aim is to collect in-depth information from the respondent. - The left-hand column lists the topic of interest - The right-hand column contains a list of suggested questions and probes. - It is not necessary to ask all these questions in the order listed; these provide ideas to prompt the respondent to talk about the topic of interest - Use a flexible approach and probe as necessary: add extra questions depending on the responses - You do not need to follow the order of topics below; follow the responses/flow of the conversation. |
| --- |

| **Topics** | **Possible questions and probes** |
| --- | --- |
| **Opening** | Hello, my named is…   - Read out the information sheet - Obtained informed consent |
| **Socio-demographic information** | - Age group - Gender - Village - Employment/livelihood - Languages spoken - Literacy/education (number of school years) |
| **Forest visits** | - For what reasons do you go to the forest? - What do you do there? - How far away is the forest from where you live? - Who do you go to forest with? Do you ever take any family members? - How long do you visit for each time you go? - When do they go there? How often? - Do you move around in the forest when you visit or do you stay in one place? - Do you go to different places for different reasons? Please explain… - Do you go to different places in the forest? at different times of year? - When are you at home most? What seasons? |
| **Sleeping arrangements in forests** | - Where do you sleep when you are in the forest? - Can you describe it (are there walls? overhead coverage? on the floor?) - Do others sleep in the same place? - Do you always sleep in the same place? If not. Where do you move to? Is it the same type of building? - Does everyone use a hammock or sleep under a net? - Where do you get the hammocks or nets from? How often do you replace them? Do you treat them? - What about if you go to a new place? Do you encounter other groups? |
| **Personal malaria-related experience** | - Have you had malaria before? - How did you know it was malaria? Was your blood tested? By whom? Using what? - Tell me about the last bout of malaria that you had… - When did you get ill? - Who diagnosed the disease? - Where do you think you got malaria from in the past? - Where did you go for treatment? - What were your experiences of taking malaria drugs? - Can you tell me where you obtained / got the drugs? What were the drugs called? |
| **Malaria-related knowledge** | - What do you know about malaria? - How is the disease caused? - Do you think anything you do puts you at risk of getting malaria? - Do you know of any places in the area where there is a high risk of malaria? - Do you think it’s possible to be infected with malaria but not have any symptoms? - How did you distinguish malaria from other diseases? - How did you get your information about malaria? - Have you heard about different types of malaria? What can you tell me about that? - If you thought you might have malaria, where would you go for help? Where else? And if it was your child? - Do you know the VMW in your village? - Where does he/she live? - Where are the nearest health centre and hospital? - Did you / a family member have to attend the health centre and/or hospital? - If yes – what for? - How can you protect yourself from catching the disease? What do you use? Why? |
| **Personal protection** | What do use to prevent from getting malaria when you are in the forest? (unprompted)  Prompted:   1. Insect repellent? 2. Coils? 3. Bednet? 4. Hammocknet? 5. Fire? 6. Antimalarials? 7. Other (specify)? |
| **Acceptability of prophylaxis** | |
| **Knowledge of the study** | - What do you know about the study that you participated in? - What disease was involved? - What happened during the study? - Who gave you the medicine? - Why were they giving you medicine? - Did everyone receive the same medicine? - Why were you chosen to participate? - How did you find out about the study? |
| **Affective attitude** | - What did you think about the malaria prevention study? - What was your opinion of the medicine that they gave? - Why did you join the study? - What did you like about the study? - What did you dislike about the study? - Would you participate again in another round of the study? - What did you think about taking medicine when you weren’t sick? - Did anyone else have any complaints about the study? What did they say? - If you were to take this medicine in future to prevent from getting malaria, where would you prefer to get it from? - For how long would you be willing to take medicine to prevent from getting malaria if it was once a week? Once a month? |
| **Perceived burden** | - Did you have problems taking the medicine? - How did you feel after taking the medicine? - Would you take it again? |
| **Perceived effectiveness** | - Did you feel that taking the medicine made you feel better or worse? - Did you feel that the medicine protected you from malaria? |
| **Fit with values and preferences** | - What are your biggest health concerns for yourself? - What are your biggest health concerns for your children? - Did you do anything different during the trial…For example, use a hammock net less? Not use repellent? Not wear long sleeved clothes? - Did any other “colleagues” who go to the forest choose not to participate? Why not? |
| **Opportunity costs** | - Did taking the medicine influence your daily routine? If so, how? - Did it influence your work in the forest? If so, how? |
| **Adherence** | - How many times did you take the medicine? - Did you miss any doses? How many? If so, why? - Did anyone you know miss any doses? Do you know why? - Did everyone in the study take all his or her doses? - Did anyone ask you to share your medicine? Why? - Why did you take the medicine? |
| **Closing** | - Do you have any questions? |
