# Supplementary material for: Acceptability and feasibility of malaria prophylaxis for forest goers: findings from a qualitative study in Cambodia
Source: Malar J. 2021 Nov 25;20:446. doi: 10.1186/s12936-021-03983-w (PMC8613728; doi:10.1186/s12936-021-03983-w)
Supplement: Supplementary file 3 — Additional file 3. Trial staff IDI guide. [file 12936_2021_3983_MOESM3_ESM.docx]

**In-depth interview guide: PAL trial staff**

| **Instructions:**   - Follow the informed consent procedures - If consent is given, audio record the interview - This interview guide is to be used in a flexible manner. - The aim is to collect in-depth information from the respondent. - The left-hand column lists the determinants that influence the implementation of prophylaxis - The middle column explains the determinant - The right-hand column contains a list of suggested questions and probes. - It is not necessary to ask all these questions in the order listed; these provide ideas to prompt the respondent to talk about the topic of interest - Use a flexible approach and probe as necessary: add extra questions depending on the responses you hear. - You do not need to follow the order the topics as presented below; follow the responses and the flow of the conversation. - Above, all show interest in the respondent and the answers that he or she gives |
| --- |

| **Topic** | **Questions** |
| --- | --- |
| **Background professional information** | - What is your current role? - Where do you currently work? - How long have you worked in this role? - Why did you decide to become a …? - Where did you work in the past? - What were your previous roles? - What professional training have you done in the past? |

| **Determinant** | **Description** | **Suggested questions/prompts** |
| --- | --- | --- |
| **Knowledge** | Degree to which the user has the knowledge needed to implement prophylaxis | Are you familiar with the trial  What do you know about it? |
| **Awareness of content of innovation** | Degree to which the user has learnt about the content of prophylaxis | What training did you receive about the programme?  What did you learn? |
| **Procedural clarity** | Extent to which the trial is described in clear steps / procedures | Are the activities that you should perform clearly defined?  Has the order in which they must be performed been clearly defined?  What is not clear? |
| **Complexity** | Degree to which implementation of prophylaxis is complex | Is the prophylaxis too complex to implement?  If too complex, what makes it too complex? |
| **Observability** | Visibility of the outcomes for the user, for example whether the outcomes of a particular treatment are clear to the user | Are the outcomes of using prophylaxis clearly observable?  What are the outcomes? |
| **Personal benefits/drawbacks** | Degree to which using prophylaxis has advantages or disadvantages for the staff themselves. | Does the programme have personal benefits/drawbacks for you?  If so, what are they? |
| **Self-efficacy** | Degree to which the user believes he or she is able to implement the activities involved in PAL | Could you, provided with the necessary support, put the different elements of the programme into practice?  If yes, what support would you require?  If not, which elements and why not? |
| **Performance feedback** | Feedback to the user about progress with prophylaxis | Do you receive feedback about progress with the implementation of the programme?  How do you receive feedback and from whom? |
| **Outcome expectations** | Perceived probability and importance of achieving the client objectives of prophylaxis | What are the objectives of prophylaxis?  Do you see these as important? |
| **Relevance for client** | Degree to which the user believes prophylaxis is relevant for his/her client. | Is the trial relevant for your clients?  If not, why not? |
| **Client satisfaction** | Degree to which the user expects clients to be satisfied with prophylaxis | Are clients satisfied or not with the intervention?  Why is this the case? |
| **Client cooperation** | Degree to which the user expects clients to cooperate with prophylaxis | Do clients cooperate with the trial?  If not, how do they resist and why? |
| **Closing** |  | Do you have any questions?  Are there any issues that you would like to raise / any ideas you would like to talk about? |
