# Supplementary material for: Acceptability and feasibility of malaria prophylaxis for forest goers: findings from a qualitative study in Cambodia
Source: Malar J. 2021 Nov 25;20:446. doi: 10.1186/s12936-021-03983-w (PMC8613728; doi:10.1186/s12936-021-03983-w)
Supplement: Supplementary file 5 — Additional file 5. Community leader IDI guide. [file 12936_2021_3983_MOESM5_ESM.docx]

**Interview guide for community leaders**

| **Instructions:**   - Follow the informed consent procedures - If consent is given, audio record the interview - This interview guide is to be used in a flexible manner. - The aim is to collect in-depth information from the respondent. - The left-hand column lists the topic of interest - The right-hand column contains a list of suggested questions and probes. - It is not necessary to ask all these questions in the order listed; these provide ideas to prompt the respondent to talk about the topic of interest - Use a flexible approach and probe as necessary: add extra questions depending on the responses - You do not need to follow the order of topics below; follow the responses/flow of the conversation. |
| --- |

| **Topics** | **Possible questions and probes** |
| --- | --- |
| **Opening** | Hello, my named is…   - Read out the information sheet - Obtained informed consent |
| **Socio-demographic information** | - Age group - Gender - Village - Role as a community leader - Years as a leader - Languages spoken - Literacy/education (number of school years) |
| **Forest going in the area** | - For what reasons do people in this area go to the forest? - What do they do there? - How far away is it? - Who do they go to forest with? Do they ever take any family members? - How long do they visit for each time you go? - When do they go there? How often? - Do they go to different places for different reasons? Please explain… - Do they go to different places at different times of year? - When are they at home most? What seasons? - Do you go to the forest? |
| **Knowledge of malaria in the area** | - Tell me about the malaria situation in this area… - Is the disease common? - Who experiences the disease most often? - Where is the disease most common? - Are there areas where lots of people contract the disease? - Do you think it’s possible to be infected with malaria but not have any symptoms? - How did you get your information about malaria? - Have you heard about different types of malaria? What can you tell me about that? |
| **Local malaria prevention** | - How do people protect themselves from malaria in this area? - Do they use nets? - Are they impregnated? - Hammock nets? - Repellents? - If not, why not? - What do you recommend people to do? - Where do people purchase nets or repellents? |
| **Chemoprophylaxis** | - What do you think about giving medicines to prevent people from getting malaria? - What would be your concerns about this as a strategy? - How long do you think people would be willing to take medicine to prevent malaria if they have to take it every week? Every month? |
| **Closing** | - Do you have any questions? |
