# Supplementary material for: Acceptability and feasibility of malaria prophylaxis for forest goers: findings from a qualitative study in Cambodia
Source: Malar J. 2021 Nov 25;20:446. doi: 10.1186/s12936-021-03983-w (PMC8613728; doi:10.1186/s12936-021-03983-w)
Supplement: Supplementary file 6 — Additional file 6. Policymaker IDI guide. [file 12936_2021_3983_MOESM6_ESM.docx]

**Interview guide for Policymakers**

| **Instructions:**   - Follow the informed consent procedures - If consent is given, audio record the interview - This interview guide is to be used in a flexible manner. - The aim is to collect in-depth information from the respondent. - The left-hand column lists the topic of interest - The right-hand column contains a list of suggested questions and probes. - It is not necessary to ask all these questions in the order listed; these provide ideas to prompt the respondent to talk about the topic of interest - Use a flexible approach and probe as necessary: add extra questions depending on the responses - You do not need to follow the order of topics below; follow the responses/flow of the conversation. |
| --- |

| **Topics** | **Possible questions and probes** |
| --- | --- |
| **Opening** | Hello, my named is…   - Read out the information sheet - Obtained informed consent |
| **Socio-demographic information** | - Age group - Gender - Country - Role - Educational/professional background |
| **National malaria prevention, control and elimination** | - What do you see as the main challenges for malaria prevention, control and elimination in your country? - Are there particular geographic areas that should be targeted? - Are there particular population groups that should be targeted? - How can existing programmes be improved? - Do you need more data on prevalence or incidence? Where is it lacking? How can it be improved? - What new tools or strategies are needed? - What type of research is needed? - What do you think about use of molecular tools (e.g. genome sequencing, PCR) in the control and elimination of malaria? In what situations would they be helpful? |
| **Addressing forest malaria** | - What are the best strategies to address forest malaria? - Is forest malaria important in your country? If so, where? - Are there any problems with current strategies for forest malaria? How can they be improved? - What research is needed? - Are there problems with implementing the strategies? |
| **Prophylaxis for forest goers** | - What do you know about prophylaxis for forest goers? - Do you see this as a way to prevent malaria in forest goers? - Do you think that it could play a role in elimination? - What are the challenges? - What type of evidence would you need to incorporate this approach in the national malaria control programme? |
| **Closing** | - Do you have any questions? |
